# Supplementary material for: Epidemiological and virological factors determining dengue transmission in Sri Lanka during the COVID-19 pandemic
Source: PLOS Glob Public Health. 2022 Aug 3;2(8):e0000399. doi: 10.1371/journal.pgph.0000399 (PMC10021909; doi:10.1371/journal.pgph.0000399)
Supplement: Attachment — Submitted filename: Answer to reviewer comments 22.05.2022.docx [file pgph.0000399.s002.docx]

May 2022

Prof. Everton Falcão de Oliveira,

Academic Editor

PLOS Global Public Health.

Dear Prof. Falcão de Oliveira,

**Submission of a manuscript titled ‘Epidemiological and virological factors determining dengue transmission in Sri Lanka during the COVID-19 pandemic**

We wish to thank the reviewers for carefully going through our manuscript and the useful suggestions they have made. We have answered their questions below and highlighted all the changes that were made in the revised version of the manuscript.

**Reviewer #1: Thank you for your important report. The article is well written, and the results do support the conclusion. This work differs from others, including serotype analyses and vector index analyses, demonstrating that social distancing measures may impact dengue transmission regardless of the virus serotype predominance and vector density.**

**Response:** We thank the reviewer for taking the time to review our manuscript and their valuable feedback

**Reviewer #2: This study aims to determine the association between the case numbers in Colombo with school closures, stringency index, changes in dengue virus (DENV) serotypes, and vector densities. Overall, this is an important study, but some points need to be improved and clarified.

1. The authors did not provide the line number on the manuscript. It would be very helpful for the reviewer if there were line numbers.**

**Response:** We apologize for the inconvenience. We have corrected this in the revised manuscript. **2. Page 2: In the abstract, you mentioned that this research aims to study the contribution of virological factors, human mobility, school closure, and mosquito factors in affecting the changes in dengue transmission in Sri Lanka. In the methods, you mentioned the stringency index. What is the relationship between the stringency index and human mobility? I would suggest defining stringency measures! What are the indicators of the stringency index?
Response**: We thank the reviewer for this comment. The stringency index as described in Our World in Data (<https://ourworldindata.org/metrics-explained-covid19-stringency-index>), is a record of how strict a country’s policies are with regard to nine indices namely: workplace closures, cancellation of public events, restrictions on public gatherings, closures of public transport, stay-at-home requirements, public information campaigns, restrictions on internal movements and international travel controls. These are all human mobility indices. We have included an explanation regarding the stringency indices in the revised version of the manuscript.

**3. Page 2 under Methods and findings: p=<0.0001. It should be p <=0.0001 or p 0.001**

**Response:** Thank you, we have made this correction in the methods section of the revised manuscript. We apologize for this error.

**4. In Introduction section, please provide more explanation regarding the important of this study and the novelty of this study.**

**Response:** We thank the reviewer for this comment. We have included this in the revised version of the manuscript in the Introduction section.

**5. Page 3: …mobility[6]. Please put a space!**

**Response:** We thank the reviewer for the feedback**.** The corrections have been made. We apologize for these inconsistencies.

**6. Page 5: …DenSys[8]. Please put a space!**

**Response:** We thank the reviewer for the feedback**.** The corrections have been made. We apologize for these inconsistencies.

**7. Page 6: … DenSys[8]. Please put a space!**

**Response:** We thank the reviewer for the feedback**.** The corrections have been made. We apologize for these inconsistencies.

**8. Page 6: … Our World in Data[11]. Please put a space!**

**Response:** We thank the reviewer for the feedback**.** The corrections have been made. We apologize for these inconsistencies.

**9. Page 6: … December 2021[12]. Please put a space!**

**Response:** We thank the reviewer for the feedback**.** The corrections have been made. We apologize for these inconsistencies.

**10. Page 7 lines 1 -11: Please revise all the formulas!**

**Response:** We thank the reviewer for the feedback**.** We apologize for the lack of clarity of the formulae. These have been revised in the manuscript.

**11. Page 7: Please define CDC as it is first mentioned**

**Response:** We thank the reviewer for the feedback**.** This has been revised. We apologize for these inconsistencies.

**12. Page 7 under the Real Time qPCR….: …DENV[13]. Please put a space!**

**Response:** We thank the reviewer for the feedback**.** The corrections have been made. We apologize for these inconsistencies.

**13. Page 7: Please be consistent in writing DEN 1-4 or DENV, DENV1, DENV2, DENV3**

**Response:** We thank the reviewer for the feedback**.** The corrections have been made. We apologize for these inconsistencies. **14. Page 8: Under Statistical Analysis:
Please complete the statistical analysis so that it covers all statistical analyses used in this study. How did you analyze the variable of school closures and human mobility? It is not mentioned in the statistical analysis**

**Response:** We used Spearman rank order correlation coefficient to analyze the relationship between dengue case numbers and the different variables. This has been corrected in the Methods section of manuscript. We apologize for the omission.

**15. Page 8: DEN 1, DEN2, DEN 3. Please be consistent!**

**Response:** We apologize for this inconsistency. We have made the correction in the manuscript.

**16. Page 10: “Sri Lanka had a stringency index over 75% in 11/21 months from April 2020 to December 2021 (Figure 3A).” What does it mean?**

**Response**: We thank the reviewer for this comment. The stringency index records 9 human mobility related indices and a score is given from 0-100%, with 100% being complete movement restriction. Sri Lanka had a stringency index over 75%, which would be considerable mobility restriction for the public, for half the time between April 2020 to December 2021.

**17. Page 10, last sentence: …and partially closed for 22 weeks, amounting to a total of 71 weeks of some form of school closure. What do you mean by partially closed? Why did it happen?**

**Response**: Partial school closure refers to either only some areas had schools open, or only children of certain grades attended school**.** We have included an explanation of this in the revised manuscript.
 **18. Page 11: “The school closures significantly correlated with a change in dengue cases in 2021 (Spearman correlation coefficient; r=-0.4732, p=<0.0001”. What does it mean? How do you interpret it? Please explain more!**

**Response:** We thank the reviewer for this comment. Dengue cases negatively correlated with school closures and that was significant. We believe that schools might be primary sources of dengue infection and thus closure of schools may have been important in driving down dengue cases numbers.

**19. Page12 line 1: ..as possible[17]. Please put a space!**

**Response:** We apologize for this, and we have corrected this in the manuscript. **20. I would suggest including some limitations of this study and some potentials future works.**

**Response:** We thank the reviewer for this suggestion**.** We have added the study imitations and future work we hope to undertake to the Discussion section of the revised manuscript. **21. Figure 2A: what does the x-axis represent?**

**Response:** We apologize for the figure being unclear. The x-axis represents the time-point at which the different serotypes percentages were recorded. We have updated the figure. **22. Please increase the resolution of Figure 3A!.**

**Response:** We apologize for the poor quality of the figure. This has been updated.
 **23. How do you interpret Figure 4A? You define the “school closure status of 1, 2 and 3, but you did not define 4.**

**Response:** We thank the reviewer for this comment. In this figure we plotted dengue case numbers vs school closure status was classified as 1=fully open, 2=partially open and 3= fully closed/academic break. The 4 was an error on our part which has now been corrected in the figure. We apologize for this mistake. **24. Please increase the resolution of Figure 5!**

**Response:** We apologize for the poor quality of the figure. This has been updated.

Thank you for considering our manuscript with PLoS Global Public Health.

Yours Sincerely,

Prof. Neelika Malavige
